# The genetic structure of *Squalidus multimaculatus* revealing the historical pattern of serial colonization on the tip of East Asian continent

**DOI:** 10.1038/s41598-018-28340-x

**Published:** 2018-07-13

**Authors:** Hyung-Bae Jeon, Dong-Young Kim, Yoon Jeong Lee, Han-Gyu Bae, Ho Young Suk

**Affiliations:** Department of Life Sciences, Yeungnam University, 280 Daehak-ro, Gyeongsan, Gyeongsangbuk-do 38541 South Korea

## Abstract

Separated river systems could create confluences via two geological processes, estuary coalescence in response to decreasing sea levels and headwater capture, allowing primary freshwater species to disperse across rivers. *Squalidus multimaculatus*, is an endemic and primary freshwater species restricted to the southeast coast of the Korean Peninsula. The distribution of this species is unique, given that other congeneric species, including its closely related *S*. *gracilis majimae*, as well as other cyprind species are observed throughout the peninsula except for the east coast. Phylogeographic analyses were conducted using three mitochondrial loci to identify the origin of *S*. *multimaculatus* and the historical pathways of dispersal. A strong phylogenetic affinity between *S*. *multimaculatus* and *S*. *g*. *majimae* and the genetic structure among populations indicated that *S*. *multimaculatus* originated from the eastward colonization of the common ancestor between *S*. *g*. *majimae* and *S*. *multimaculatus* via headwater capture through fault zones within successive mountain range. Following colonization, the ancestral *S*. *multimaculatus* likely migrated towards north via estuary coalescence along a well-developed continental shelf. Our study was the first empirical attempt providing insights into how freshwater organisms dispersed to the southernmost tip of East Asia, despite the potential loss of such historical imprints with anthropogenic interference.

## Introduction

In freshwater ecosystems, primary freshwater fish species cannot naturally move to other physically separated drainages, and landscape structures may even become barriers within a single drainage, limiting gene flow among populations^1,2^. Because of this fragmented nature, freshwater ecosystems can provide exceptional opportunities for analysis of the contribution of physical isolation to intraspecific genetic structure^1,3,4^. By combining the genetic data of intraspecific population structures with information regarding historical drainage structures and geological events, it is possible to infer how a freshwater species or its ancestor have dispersed across rivers shaping the present distribution^2,4–6^.

With time, separated river systems could create confluences via two representative geological processes, allowing freshwater species to disperse across rivers that are not currently connected^7,8^. First, the estuary regions of adjacent rivers were often connected in response to decreasing sea levels during the Quaternary period (estuary coalescence)^9–11^, though connections could rather rarely occur between rivers that did not share the same well-developed continental shelf^12,13^. This geological phenomenon seems to have occurred in regions with wide continental shelves, where rivers in geographical proximity are likely to share estuaries^8,10,14^. Second, the uppermost region of a river may be diverted from its own bed and merged with the headwater of other adjacent rivers (headwater capture)^11,15,16^. Although headwater capture is still observable with watershed erosion in a specific condition^17^, it is a geological phenomenon that frequently occurred via strong tectonic deformation along fault zones^17–19^.

**Figure 1:**
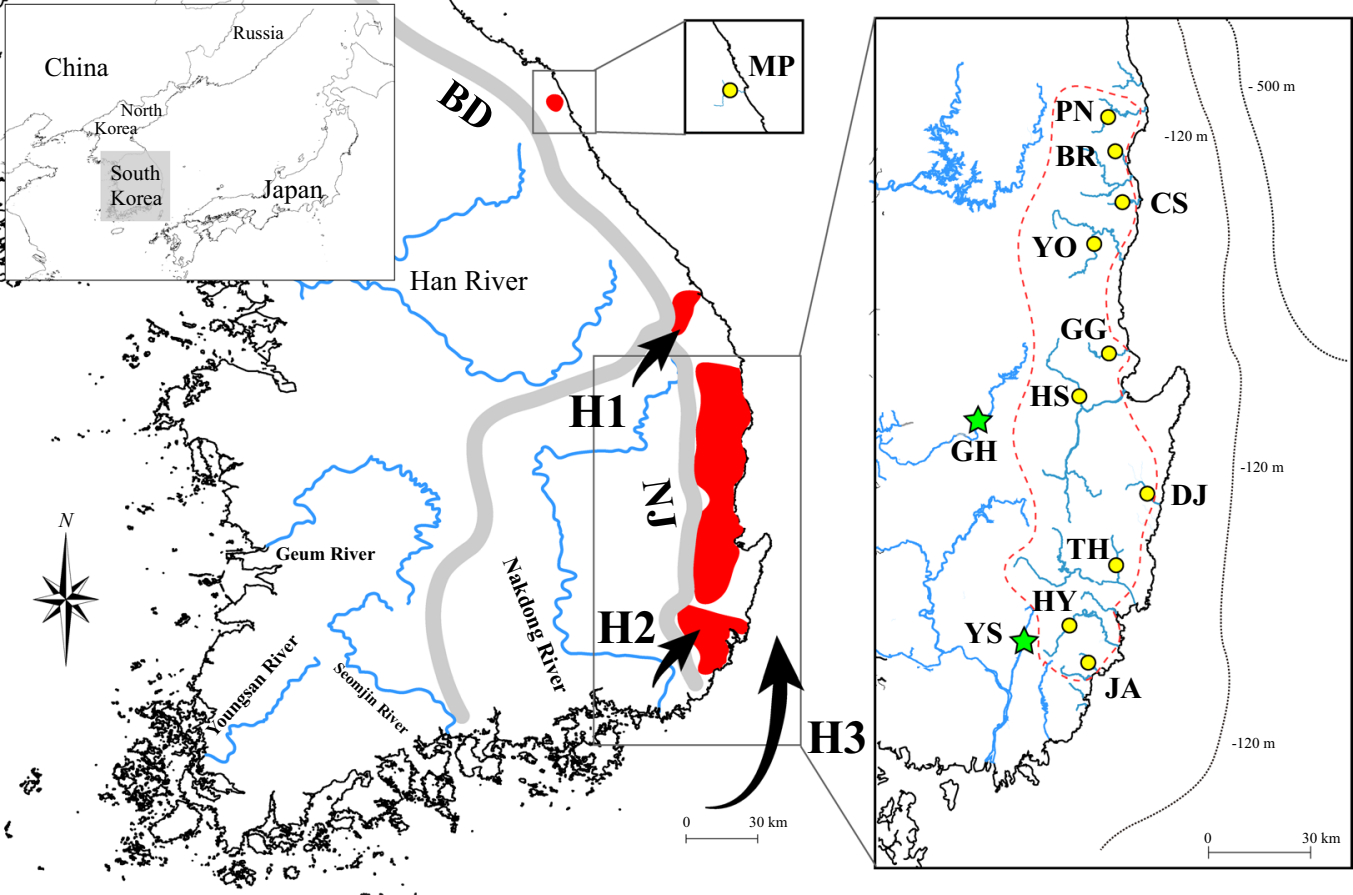
The distribution range of *Squalidus multimaculatus* (red-colored areas) and *S*. *gracilis majimae* (major river systems flowing the south and west coasts). The collection sites of *S*. *multimaculatus* (yellow circles) and *S*. *g*. *majimae* (green stars) are shown in the squares positioned on the right and middle top (see Table S1 for the detailed information). Arrows indicate the three hypothetical routes (H1, H2 and H3)^16,26,28^ of ancestral *S*. *multimaculatus* through successive mountain range (BD: Baekdudaegan; NJ: Nakdongjeongmaek). The map was generated by Adobe Illustrator CC 2015 using a GIS shape file retrieved from GADM database (www.gadm.org; v 2.5; freely available for non-commercial purposes) in DIVA GIS (http://www.diva-gis.org/) and modified in QGIS v 2.16.3 in accordance with the guidelines suggested in the websites.

The Korean Peninsula is a small area located at the end of the East Asian continent (Fig. 1). The Yellow Sea, located between the peninsula and western China (Fig. 1), was part of the Yellow River, a major river system flowing east through China, when the sea level was lower than at present during the last glacial maximum (LGM)^6,20,21^. The Yellow Sea probably created confluences with the present-day major river systems along the south and west coast on the Korean Peninsula^21–23^. Conversely, the east coast of this peninsula is geographically isolated by successive mountain ranges (Fig. 1); accordingly, the freshwater ecosystems on the east coast likely had confluences with a different large drainage system (i.e., the Paleo-Amur River)^24,25^. Despite the landscape isolation, the freshwater ecosystems along the southern east coast might have historically been coalesced with the Paleo-Yellow Sea^9,21,25,26^. However, no empirical approach has been conducted to date to reconstruct colonization processes around this area.

The spotted-barbel gudgeon, *Squalidus multimaculatus*, is an endemic freshwater species (Cyprinidae) restricted to the southern east coast of the Korean Peninsula^25,27^. The distribution of this species is somewhat unique, given that other congeneric species (*S*. *gracilis majimae*, *S*. *chankaensis tsuchigae* and *S*. *japonicus coreanus*) are observed in most freshwater systems throughout the peninsula except for the east coast^25,27^. Among these congeneric species, *S*. *g*. *majimae* is morphologically and genetically close to *S*. *multimaculatus*^28^. Thus, it is conceivable that *S*. *multimaculatus* originated from the migration of the common ancestor of *S*. *multimaculatus* and *S*. *g*. *majimae* individuals inhabiting the drainage areas close to the east coast such as the Nakdong River, the longest river flowing to the south coast in South Korea^28^. Three hypothetical routes for this geodispersal process could be envisaged based on the geographical features on the east coast and previous studies (Fig. 1)^16,26^. There might have been headwater confluences from the tributaries of the Han (second longest river flowing to the west coast) and Nakdong River to the Samcheok-Osip River (H1)^16^ or directly to rivers on the southern east coast (H2; Fig. 1)^26,28^. The second hypothesis (H2) was established based on the existence of Yangsan Fault Zone between the Nakdong River and the southern east coast and its tectonic history. Considering the geographic proximity, the mouths of the Nakdong River (or adjacent small streams) and southernmost east coastal rivers likely coalesced following the Quaternary climate changes (H3; Fig. 1), though this hypothesis has yet to be validated empirically.

In this study, three mitochondrial loci were used to analyze the phylogenetic relationship among the populations of *S*. *multimaculatus* and *S*. *g*. *majimae* (Supplementary Table S1). In addition, divergence ages among phylogenetic groups of *S*. *multimaculatus* and *S*. *g*. *majimae* were estimated for comparison with the history of geological changes in the landscape structures isolating the east coast. These analyses could allow us to identify the most valid one among the three biogeographic routes presented above for the colonization of *S*. *multimaculatus*. These data were also used to reconstruct the historical migration pattern of *S*. *multimaculatus* following its colonization within the east coast by analyzing the demographic histories of the phylogenetic groups. Although this study is a biogeographic investigation of a single species, our results are anticipated to provide critical insight into the historical pattern of drainage formation and freshwater dispersal on the tip of East Asian continent.

## Results

The primers used in this study successfully amplified all individuals. A total of 16, 61 and 26 haplotypes were detected from the sequences of COI (670 bp), cyt *b* (1,128 bp) and 12*S* (949 bp), respectively. Among these, *Squalidus gracilis majimae* contained 5, 23 and 9 haplotypes in COI, cyt *b* and 12S, respectively. Except for two populations, JA and YO, no haplotypes were shared between these two species (Supplementary Tables S2–S4). COI, cyt *b* and 12*S* contained 41 (6.1%), 137 (12.1%) and 34 (3.5%) polymorphic sites, and 29 (4.3%), 104 (9.2%) and 21 (2.2%) parsimonious informative sites, respectively.

**Figure 2:**
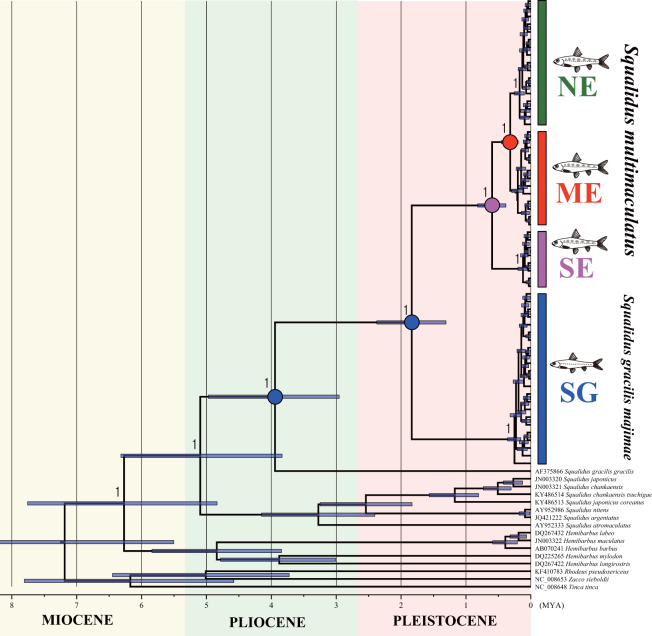
Time-calibrated Bayesian tree reconstructed by BEAST using cyt *b* sequences. The 95% highest posterior confidence intervals of divergence times are indicated on every major node with blue bar. Ancestral area reconstruction was also inferred based on the Bayesian Binary Markov Chain Monte-Carlo (BBM) and incorporated in the tree. The color circle of nodes showed the most likely ancestral area; blue: western Korean Peninsula, pink: Taehwa and Hoeya River (region SE), red: Hyeongsan River (region ME).

All three algorithms (NJ, ML and BI) generated highly concordant tree topologies in the phylogenetic analysis reconstructed based on cyt *b* sequences (Supplementary Fig. S2). *S*. *g*. *majimae* haplotypes (SG) were resolved as the most likely sister group of *S*. *multimaculatus*, and Japanese *S*. *gracilis* was placed as the sister to the cluster of *S*. *g*. *majimae* and *S*. *multimaculatus* (Supplementary Fig. S2). Two other congeneric species inhabiting the Korean Peninsula, *S*. *japonicus coreanus* and *S*. *chankaensis tsuchige*, were somewhat separated from *S*. *g*. *majimae* and *S*. *multimaculatus* in the tree (Supplementary Fig. S2). Haplotypes of *S*. *multimaculatus* were likely to be allocated into three haplogroups, though the distinction of ME and NE was not clear in the ML and BI trees (Supplementary Fig. S2). The name of haplogroups were assigned as NE (northeast), ME (mid-east) and SE (southeast) in accordance with the geographic regions in which they were predominantly observed (Supplementary Tables S2–S5), which was also supported by the result of AMOVA (Tables S6). The ME and NE haplogroups formed a cluster, while SE was placed as a sister to that cluster (Supplementary Fig. S2). In the phylogenetic tree analysis using the other two loci (COI and 12*S*), the haplogroup SE was clearly isolated from others, but the distinction between ME and NE was somewhat difficult (Supplementary Figs S3–S4). The divergence between *S*. *g*. *majimae* and *S*. *multimaculatus* was estimated to be 1.83 (95% HPD 0.31–2.37) MYA (Fig. 2). Within *S*. *multimaculatus*, age estimates of 0.59 (95% HPD 0.38–0.81) MYA and 0.31 (95% HPD 0.19–0.45) MYA were allocated to the nodes leading to the separation of haplogroup SE and the divergence between NE and ME, respectively (Fig. 2). Our analysis of ancestral area reconstruction (BBM) showed that the common ancestor of both *S*. *g*. *majimae* and *S*. *multimaculatus* was inferred to be distributed in the southwestern Korean Peninsula and that the common ancestor of *S*. *multimaculatus* colonized in SE region for the first time before dispersion (Fig. 2).

Based on our NJ analysis results, each population was included in a single haplogroup. Six northern populations (MP, PN, BR, CS, YO and GG; NE region) only contained NE haplotypes in the three loci, except that a single SE haplotype of cyt *b* and a few haplotypes of *S*. *g*. *majimae* were found in population YO (Supplementary Tables S2–S5). Population HS (mid-east region) contained haplotypes allocated to haplogroup ME, whereas SE haplotypes were predominantly found in populations TH and HY (southeast region), though one ME haplotype in both COI and cyt *b* was found in population TH (Supplementary Tables S2–S5). Population DJ contained haplotypes that could be allocated to haplogroup NE, despite its geographical proximity to populations HS and TH (Supplementary Tables S2–S5). The haplotype composition in population JA was somewhat complicated because the haplotypes of ME and *S*. *g*. *majimae* coexisted (Supplementary Tables S2–S5). Analysis of RAG1 sequences (1,455 bp) based on the double peak calling of both species specific nucleotides revealed that twelve individuals in population JA and three individuals in population YO were hybrids of *S*. *multimaculatus* and *S*. *g*. *majimae* (Supplementary Tables S2–S5; Fig. S1).

**Figure 3:**
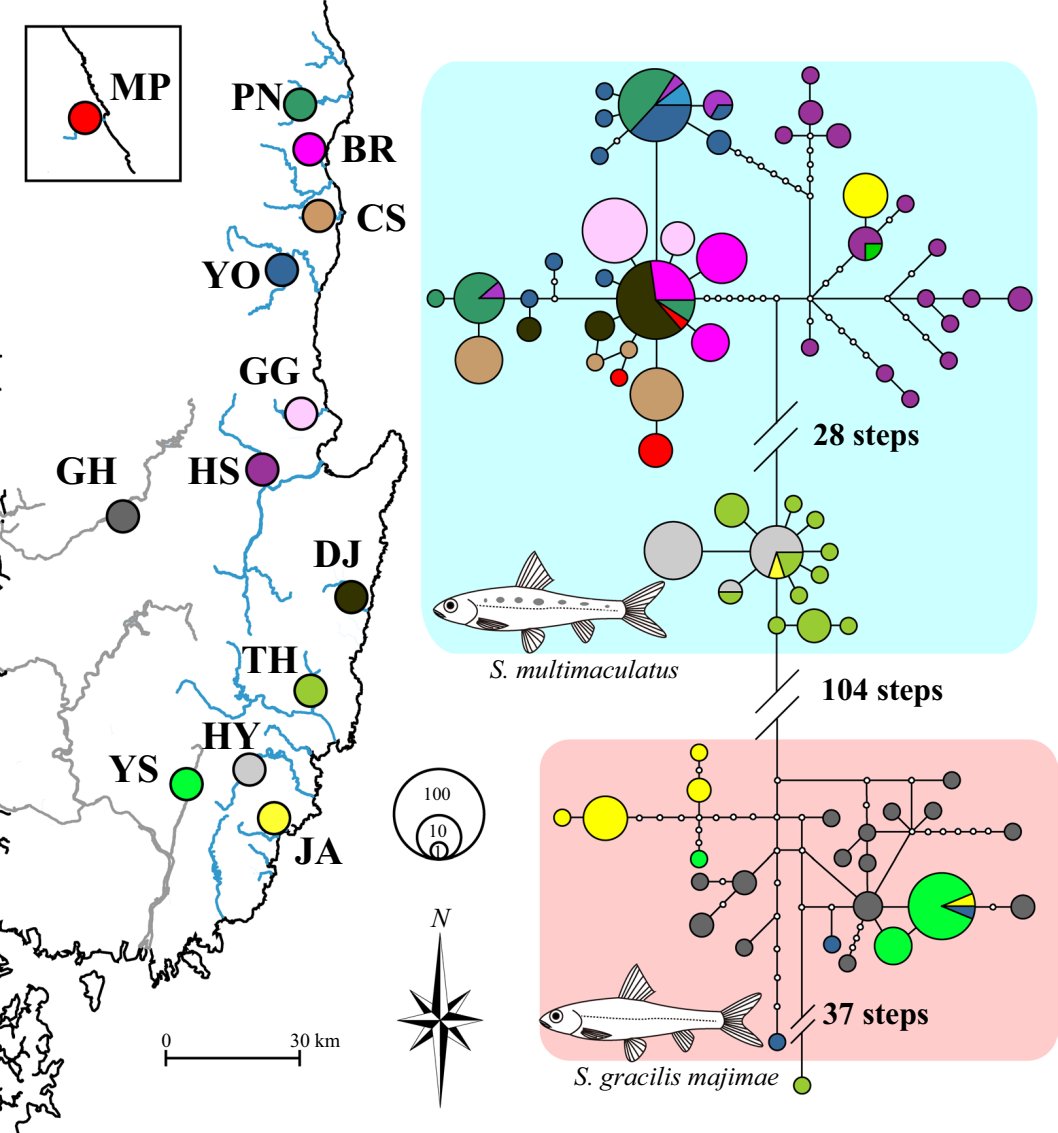
The unrooted network generated based on the haplotypes of three mitochondrial loci (COI, cyt *b* and 12S) from *Squalidus multimaculatus* and *S*. *gracilis majimae*. See Fig. 1 for the details about how to generate the map.

The presence of three haplogroups was also evident upon network analysis with the combined datasets of the three mitochondrial loci (Fig. 3). In this analysis, haplogroups NE and ME were genetically close to each other, similar to the results of phylogenetic tree analyses, whereas haplogroup SE appeared to be closer to the haplotypes of *S*. *g*. *majimae* than the other two haplogroups (Fig. 3), indicating that the first founder of *S*. *multimaculatus* likely colonized the SE region. Network analysis with cyt *b* revealed a similar pattern of separation among the three haplogroups (Supplementary Fig. S5). However, the separation of haplogroup SE was only observed upon analysis with 12*S*. In addition, no noticeable separation was observed upon analysis with COI, despite a slight difference between NE and ME (Supplementary Fig. S5).

The intra-population genetic diversity was estimated and compared based on the combined dataset of three mitochondrial loci (Table 1; see also Supplementary Tables S7–S9). Populations YO (NE), HS (ME) and TH (SE) exhibited the highest level of genetic diversity among *S*. *multimaculatus* populations (Table 1). The remaining NE populations (MP, PN, BR, CS, GG and DJ) showed low intra-population genetic diversities (Table 1). The genetic diversity of population HY (SE) was also relatively low (Table 1). A moderate level of diversity was observed in population JA (Table 1). For *S*. *g*. *majimae*, population GH was higher than that of YA (Table 1).

**Table 1:** The genetic diversity indices of the *Squalidus multimaculatus* and *S*. *gracilis majimae* estimated based on the combined dataset of three mitochondrial loci (COI, cyt *b* and 12S).

Pop (haplogroup)	*N*	*π*	*h*	*h*_*d*_	*S*	Tajima’s *D*	Fu’s *F*s	SSD
MP (NE)	9	0.00101	5	0.806	9	−0.741	−0.122	0.055
PN (NE)	20	0.00098	4	0.932	6	1.910	2.793	0.145
BR (NE)	20	0.00033	3	0.679	2	1.464	1.020	0.006
CS (NE)	20	0.00044	4	0.616	8	1.624	3.627	**0**.**238**
YO (NE)	20	0.01328	13	0.884	140	−0.312	3.784	0.027
GG (NE)	20	0.00027	3	0.416	2	0.698	0.534	**0**.**282**
HS (ME)	20	0.00239	15	0.968	31	−0.970	**−4**.**869**	0.013
DJ (NE)	20	0.00044	4	0.563	6	−0.895	0.452	0.031
TH (SE)	20	0.00473	13	0.932	110	**−2**.**389**	−0.044	0.024
HY (SE)	20	0.00022	3	0.542	2	0.172	0.153	0.020
JA (ME)	20	0.02283	7	0.774	140	2.434	21.035	**0**.**121**
YS	20	0.00053	3	0.468	11	**−1**.**866**	2.224	0.021
GH	20	0.00184	15	0.968	31	**−1**.**658**	**−6**.**516**	0.008

Data comprise number of individuals analyzed (*N*), nucleotide diversity (*π*), number of haplotypes (*h*), haplotype diversity *(h*_*d*_), number of segregating sites (*S*), Tajima’s *D*, Fu’s *F*s and sum of squares deviation (SSD). Statistically significant values were highlighted with bold.

**Figure 4:**
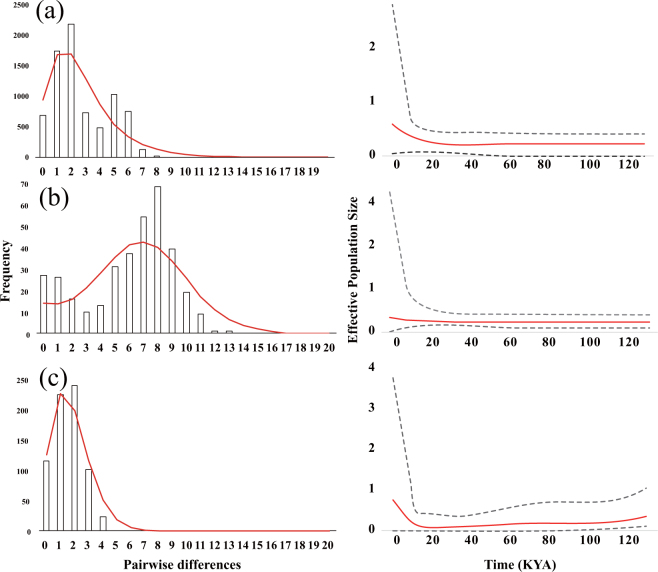
The results from the mismatch distributions (left) and extended Bayesian skyline plots (right) analyzed based on the combined dataset of three mitochondrial loci (COI, cyt *b* and 12S) in haplogroups NE (**a**), ME (**b**) and SE (**c**) of *Squalidus multimaculatus*.

Neutrality tests revealed that haplogroups NE and SE had significant negative Fu’s *F*s and Tajima’s *D* values (Table 2; see also Supplementary Tables S10–S12), indicating the signature of demographic expansion in the sizes of those haplogroups following diversification and colonization. The Tajima’s *D* and Fu’s *F*s values of haplogroup ME were negative but not significant; thus, the neutrality model could not be rejected (Table 2; see also Supplementary Tables S10–S12). Upon mismatch analyses, haplogroups NE and SE showed a clearly unimodal pattern, confirming the results based on the estimation using the Fu’s *F*s and Tajima’s *D* values (Fig. 4). A bimodal shape was exhibited in haplogroup ME, indicating that its size has been relatively stationary since colonization (Fig. 4). Our Bayesian skyline plot analyses showed that the effective population sizes of haplogroups NE and ME increased by about 100 KYA (Fig. 4). Haplogroup SE underwent gradual decline in population size and subsequent rapid increase at about 20 KYA.

**Table 2:** The comparison of genetic diversity indices among the haplogroups of *Squalidus multimaculatus* and *S*. *gracilis majimae* estimated based on the combined dataset of three mitochondrial loci (COI, cyt *b* and 12S).

Haplogroup	*N*	*π*	*h*	*h*_*d*_	*S*	Tajima’s *D*	Fu’s *F*s	SSD
NE	125	0.00134	25	0.913	84	**−2**.**440**	**−7**.**445**	0.019
ME	29	0.00218	16	0.923	32	−1.028	−3.972	0.015
SE	40	0.00117	13	0.845	44	**−2**.**450**	**−2**.**809**	0.007
SG	55	0.00198	21	0.872	76	**−2**.**466**	**−6**.**205**	0.007

Data comprise number of individuals analyzed (*N*), nucleotide diversity (*π*), number of haplotypes (*h*), haplotype diversity *(h*_*d*_), number of segregating sites (*S*), Tajima’s *D*, Fu’s *F*s and sum of squares deviation (SSD). Statistically significant values were highlighted with bold.

## Discussion

In our phylogentic analyses, *Squalidus gracilis majimae* was resolved to be the closest sister to *S*. *multimaculatus*. Although our analysis does not take into account extinct taxa, when solely comparing all of the existing species in *Squalidus*, it is conceivable that *S*. *multimaculatus* on the east coast originated from colonization by individuals of *S*. *g*. *majimae* or its derived populations. The results of our haplotype network and phylogenetic analyses showed that *S*. *multimaculatus* populations could be divided into three major haplogroups, NE, ME and SE. The NE and ME haplogroups closely clustered with each other, while SE was rather separated, but showed relatively higher affinity to *S*. *g*. *majimae*. These results, combined with past geological changes, could provide the basis for verifying which of the three hypothetical dispersal paths described in view of existing literatures and geographical environment is most plausible.

The first hypothetical route was established based on the possibility of migration from the major freshwater systems on the Korean Peninsula (south and west) to the Samcheok-Osip River on the east coast by headwater capture^16,29^. Since the Samcheok-Osip River is located somewhat northward of the known natural distribution range of *S*. *multimaculatus* (from PN to JA), it is unreasonable to regard this river as a starting point for colonization. Although *S*. *multimaculatus* individuals were observed in the Samcheok-Osip River as well as several other northern rivers (including population MP) in our field investigations and previous studies^30–32^, those individuals probably existed because of artificial transplanting given that the presence of this species has never been known until a few decades ago, despite intensive field investigations^29,30,33^. Furthermore, considering the results of our phylogenetic tree analyses that showed NE and ME were clustered together and SE was placed as a sister group, it is difficult to explain how this species could spread towards southern east coastal rivers.

**Figure 5:**
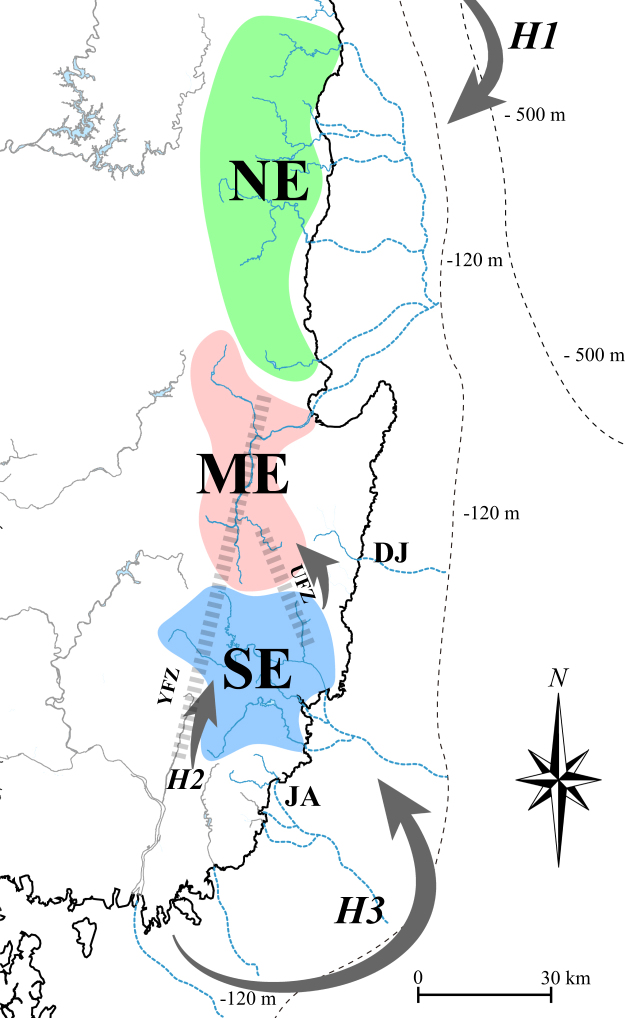
The colonization route of *Squalidus multimaculatus* inferred based on our genetic results and previous geologic studies. Dashed lines on the ocean indicate the range of the continental shelf developed along the east coast on the Korean Peninsula. Blue lines indicate the virtual paleo-drainage system existed along the coast when the sea levels were lower than at present designed to illustrate the confluences inferred. Gray arrows indicate the historical dispersal route of *S*. *multimaculatus* or its ancestors through Yangsan (H2; YFZ) and Ulsan (UFZ) Fault Zones. See Fig. 1 for the details about how to generate the map.

The second hypothetical route was established based on the fact that the Yangsan Fault Zone between the Nakdong River and rivers along the southern east coast has undergone active crustal fluctuations^34–36^, and two freshwater systems (the Nakdong River and southern east coastal rivers) were likely to have fused together during the geological changes (Fig. 5). In this fault zone, the strongest activities occurred about 1 million years ago^34–36^, which is the same time at which *S*. *g*. *majimae* and *S*. *multimaculatus* were estimated to have diverged in our analyses. The haplotypes of populations TH and HY (SE) exhibited the highest affinity to the haplotypes observed in *S*. *g*. *majimae* in our haplotype network analyses, indicating that ancestral *S*. *multimaculatus* founders colonized the SE region for the first time in the east coast. As shown in the phylogenetic clustering pattern, it is conceivable that *S*. *multimaculatus* individuals naturally migrated north from this point. This second hypothesis can also be supported by the fact that species inhabiting the Nakdong River, such as *Cobitis hankugensis* (Cobitidae), *Iksookimia longicorpa* (Cobitidae) and *Liobagrus* sp. (Liobagridae), are also present in SE regional rivers^37–39^. Although it is known that a historical migration occurred between the Hyeongsan River (population HS) and the Nakdong River through the Yangsan Fault^40^, our data did not verify this possibility.

In SE, the genetic diversity of population TH was much higher than that of HY, presumably indicating that this species (or its common ancestor) first colonized the Taehwa River (TH; Fig. 5) and then moved to the Hoeya River (HY), though the contemporary diversity estimates may not represent the past demographics. The mouths of the two rivers are geographically adjacent, and they were likely occasionally linked during the sea level fluctuation in the last glacial maxima (Fig. 5)^41–44^. This inference is also supported by the ichthyofaunal similarity between the two rivers^38,45^. Our EBSP results indicated that the dispersal within the SE region was most active around the late Pleistocene (20 KYA), because sudden demographic growth was noticeable during this period, following the gradual decline since 200 KYA. How could *S*. *multimaculatus* that colonized the SE region disperse to the river in the north? Headwater capture has also likely contributed to the migration of *S*. *multimaculatus* founders to the Hyeongsan River (population HS). The Ulsan Fault Zone is located between the Hyeongsan River and the Taehwa River (population TH), and the watersheds of both rivers are in geographical proximity (Fig. 5). The divergence time of haplogroup ME and NE from SE was estimated to be 430 KYA in our analyses, which completely coincides with the active period of this fault^35,46^. Taken together, these findings indicate that the headwater capture between those two rivers was a starting point of northward dispersal of *S*. *multimaculatus*.

Population HS exhibited an exceptionally high level of diversity in all three loci examined. In addition, haplogroup ME did not show any demographic decline or sudden fluctuations in our EBSP results. This was probably related to the physical habitat in the area inhabited by this population. Although most east coastal rivers are small and subject to weather-sensitive physical changes, even during a short-term period, the Hyeongsan River is large, conceivably providing stable and wide habitats that maintain a large-sized population. Since there is no fault zone from the Hyeongsan River towards the north, headwater capture might not have contributed to colonization in the NE region. Instead, there is a well-developed continental shelf, that is likely to have served as a place for the coalescence among river mouths. The historical northward migration from ME to NE is supported by the finding that natural populations of *Iksookimia yongdokensis* (Cobitidae) are observed in both the ME and NE region, but not the SE region^25^.

The third hypothetical route was made by the assumption that frequent estuary coalescence between south coastal rivers (including the Nakdong River) and southern east coastal rivers probably contributed to the migration of freshwater species. If this assumption is correct, the Jangan River (population JA; ME), which is located where the south and east coast meet, might have served as a bridge for the historical colonization of *S*. *multimaculatus* to the east coast. However, our results did not provide any evidence supporting such an assumption. The population JA did not exhibit the highest genetic affinity to population HY (in the Hoeya River; SE), located in the immediate north, but was closer to population HS (Hyeongsan River; ME). As discussed below, haplogroup ME did not show the highest genetic affinity to *S*. *g*. *majimae*. In addition to our results, we made an effort to find evidence of water connection around this area. No geological evidence has been known about the connection, for example between the Jangan River and any from SE region. Since the headwaters are not geographically adjacent around this area, the possible water connection might be the estuary coalescence responding to sea level fluctuation. However, the continental shelf on the coast is too narrow to imagine the effect of sea level change. Except for *S*. *multimaculatus*, there was no ichthyofaunal commonality between the Janan River and SE rivers.

In our analyses, several populations showed quite unexpected genetic characteristics. First, population DJ was part of haplogroup NE in our analyses, despite its geographical proximity to SE or ME populations, which cannot be explained solely based on the biogeographical inference. This may be the results of artificial introduction, though this prediction should be examined by empirical studies in the future. Second, as already mentioned, MP was also considered to be created by artificial introduction. In the Myeongpa River (MP), there are several cyprinid species that were not observed in other adjacent rivers, including *Zacco platypus*, *Z*. *koreanus* and *Aphyocypris chinensis*, indicating that some massive introduction might have occured^31,32^. However, the results of this study do not clearly indicate NE rivers from which this population originated. In particular, two cyt *b* gene haplotypes, NE04 and NE06, observed in population MP were not present in any of the NE populations analyzed in the present study, indicating the possibility of transplantation from the river(s) that was (were) not analyzed in this study.

Population JA contained the haplotypes of both *S*. *multimaculatus* and *S*. *g*. *majimae*. Where did the individuals of this population come from? *S*. *multimaculatus* haplotypes in this population most likely originated from population HS. Only a single ME haplotype was observed, indicating that a small number of individuals were introduced to this population probably from the population HS. A single SE haplotype was also observed in this population, which may be the evidence of additional introductions from SE regional rivers, though the possibility of natural existence could also be regarded. However, the haplotype diversity of *S*. *g*. *majimae* was somewhat higher than that expected to be formed by artificial introduction of a small number of individuals. Therefore, it is conceivable that *S*. *g*. *majimae* individuals might have existed naturally prior to the introduction of *S*. *multimaculatus* in this river. In addition, some *S*. *g*. *majimae* haplotypes in this population were not observed in two populations from the Nakdong River, indicating the possibility of introduction from other river(s). However, caution is needed when drawing conclusions regarding the historical origins of this population, because sampling has not been performed throughout the Nakdong River. A low frequency of *S*. *g*. *majimae* haplotypes was also observed in the population JA, indicating that a small number of *S*. *g*. *majimae* individuals were introduced in this population. Examination with the nuclear marker (RAG1) showed clear evidence of active hybridization between *S*. *g*. *majimae* and *S*. *multimaculatus* in both populations.

## Conclusion

In our genetic analyses, we inferred the route of colonization and distribution origin of a freshwater fish species at the southeast tip of East Asia, though further comparative studies are required to confirm this result. The first colonization of *S*. *multimaculatus* ancestral population likely occurred through confluence caused by headwater capture during geological changes in landscape structures. In the rivers around mid- and northern east coast regions where the continental shelf is wider than the southern part of the eastern coast, estuary coalescence could be presumed to be the main route of migration and colonization of this species. However, our study also showed the potential loss of such historical imprints that have been created over a million years because of anthropogenic interference.

## Materials and Methods

### Sampling and sequencing

All samples used were collected according to the Inland Water Fisheries Act and Wildlife Protection and Management Act of the Republic of Korea. The entire procedure of this study was approved by the Yeungnam University Institutional Animal Care and Use Committee (Protocol # 2015013). A total of 209 individuals of *Squalidus multimaculatus* were collected from 11 east coastal rivers on the Korean Peninsula (Fig. 1; Table 1). A total of 40 individuals of *S*. *gracilis majimae* were also collected from two spots located on the Nakdong River (Fig. 1; Table 1). Number of scales above the lateral-line was rapidly counted for all individuals collected, because this characteristic was known to be different between *S*. *multimaculatus* and *S*. *g*. *majimae*^25,27,28^. All individuals collected were released back to their original habitats after removing a small fragment of tissue from each caudal fin. All tissue samples were preserved in 99% ethanol until they were used for DNA extraction and further genetic analyses. Whole genomic DNA was extracted using a Wizard^®^ Genomic DNA Purification Kit (Promega, Madison, WI, USA).

Three mitochondrial loci, cytochrome b (cyt *b*), cytochrome oxidase subunit 1 (COI) and 12*S r*RNA (12*S*), were analyzed in this study (Supplementary Table S1). The nuclear RAG1 gene was also analyzed only for detection of hybrid individuals (by checking the existence of double peaks of *S*. *multimaculatus* and *S*. *g*. *majimae* specific nucleotides) known to occur in several *S*. *multimaculatus* populations^28^ (Supplementary Fig. S1). PCR was performed in a 25 µl mixture containing 1 µl DNA template, 1× *Taq* Buffer, 0.2 mM dNTPs, 2.5 µM of each primer and 2.5 units of *Taq* DNA polymerase (Genetbio, Daejeon, South Korea). Thermal cycling was performed under a program consisting of initial denaturation at 94 °C for 2 m, 35 cycles of 94 °C for 30 s, 54 °C (or 56 °C for COI) for 30 s and 72 °C for 30 s and then final extension at 72 °C for 10 m. Amplified products were purified with the PrimePrep PCR Purification Kit (Genetbio, Daejeon, South Korea) and sent to Genotech (Daejeon, South Korea) for sequencing, which was conducted in an ABI3730xl DNA analyzer (Applied Biosystems, Foster City, CA) with the same primer sets used in our PCR reactions. The residues of DNA samples were deposited at the Department of Life Sciences, Yeungnam University.

### Diversity estimation

All of the haplotype sequences were deposited in GenBank (Accession number: KY486515–486635). DNA sequences analyzed for all four loci (including RAG1) were initially edited using Geneious 9.1^47^, after which they were manually checked for erroneous base calls. Multiple sequence alignments were performed using ClustalW^48,49^ implemented in MEGA6^50^. Genetic diversity indices of three mitochondrial loci, including nucleotide diversity (*π*)^51^, number of haplotypes (*h*)^51^, haplotype diversity *(h*_*d*_)^51^ and number of segregating sites (*S*)^52^, were estimated using DnaSP 5.1^53^.

### Phylogenetic analyses

Phylogenetic trees were generated under three different algorithms, Bayesian inference (BI), maximum likelihood (ML) and neighbor-joining (NJ). All three mitochondrial loci were used in the reconstruction of phylogenetic tree. Since the cyt *b* sequence is the most widely reported in fish species, however, the phylogenetic tree using key species as outgroup could only be reconstructed using this locus. The outgroup species were selected from Cyprinidae as follows: five *Hemibarbus* species including *H*. *labeo* (DQ267432), *H*. *maculatus* (JN003322), *H*. *barbus* (AB070241), *H*. *mylodon* (DQ225265), *H*. *longirostris* (DQ267422), eight *Squalidus* species including *S*. *japonicas* (JN003320; Japan), *S*. *chankaensis* (JN003321; China, Mongol and Russia), *S*. *chankaensis tsuchigae* (KY486514; Korea), *S*. *japonicus coreanus* (KY486513; Korea), *S*. *atromaculatus* (AY952333; China and South Asia), *S*. *nitens* (AY952986; China), *S*. *argentatus* (JQ421222; China and South Asia) and *S*. *gracilis gracilis* (AF375866; Japan), *Rhodeus pseudosericeus* (KF410783), *Tinca tinca* (NC008648) and *Zacco seiboldii* (NC008653). Jmodeltest2^54,55^ was implemented in CIPRES portal version 3.1^56^ to identify the most appropriate nucleotide substitution models for BI and ML based on the Akaike information criterion (AIC)^57^. The best fitting model was found to be TIM3 + I + G.

BI analysis was conducted using MrBayes 3.2^58^ implemented in the CIPRES portal. Each BI analysis consisted of two parallel runs of 80 million Markov Chain Monte Carlo (MCMC) generations with sampling every 1,000 steps. The consensus tree for each data set was generated after omitting the first 25% of sampled trees as burn-in. The node confidence in the BI tree was presented with the Bayesian posterior probabilities. The convergence and stationarity of chains were determined based on whether the effective sampling size (ESS) reached more than 200 using TRACER 1.6^59^. The ML tree was created using RAxML GUI 1.5^60^ under the GTRGAMMA model with 1000 bootstrap replicates. NJ analysis was performed using MEGA6 under the Kimura 2-parameter model (K2P)^61^. Node confidence in the NJ tree was assessed based on 1,000 bootstrap replicates.

Hierarchical genetic structuring was analyzed by assessing the relative contributions of among-haplogroup, among-population and within-population components using an analysis of molecular variance (AMOVA), which was performed using Arlequin 3.5^62^. The unrooted haplotype network was initially generated for four different datasets (cyt *b*, COI, 12*S* and combined) based on the connection limit above 0.95 in probability using TCS 1.2^63^. When the link was disconnected between haplogroups, PopART^64^ was applied to generate the connection.

### Divergence time estimation

Divergence time estimation among phylogenetic groups was conducted in BEAST 2.3.0^65^ using cyt *b* sequences in the CIPRES portal. The BEAST analysis was run using a strict and an uncorrelated lognormal relaxed molecular clock to examine the variation of rate across branches. Posterior distributions of parameters were estimated using 40 million MCMC generations, with samples drawn every 1,000th steps under the model chosen as the most-fit, TIM3 + I + G. The initial 10% of samples were discarded as burn-in. The analysis was conducted using a Yule speciation and a birth-death model for the tree prior to examine the sensitivity of the results to the choice of tree prior. The genus *Hemibarbus* was known to be a sister to *Squalidus*^66^ and its fossil record (5 MYA)^67^ was available as the calibration point to constrain the ages. This record was assigned in the form of uniform prior to provide a minimum age for the separation of *Hemibarbus* and *Squalidus*. TRACER 1.6^59^ was used to confirm whether ESS exceeded 200 and the MCMC parameters of runs converged on the same stationary point with unimodal distribution. A consensus tree was generated after discarding the first 20% of trees as burn-in in TreeAnnotator 2.3.0^65^, and was visualized in FigTree 1.4.2^68^. The node credibilities of the consensus tree were evaluated by posterior probabilities. To examine dispersal scenario for *S*. *multimaculatus*, the distributional areas of common ancestor was inferred using the Bayesian binary MCMC method (BBM)^69^ implemented in RASP^70^. BEAST tree was used as an input for this analysis. Six areas are assigned based on the distributional area of the species used in the BEAST tree: (A) China; (B) Korean Peninsula excluding east coastal rivers; (C) the Taehwa and Hoeya River; (D) the Hyeongsan River; (E) the Myeongpa, Pyeonghae-Namdae, Baegrok, Chuksan, Youngdeok-Osip and Gokgang River; (F) Japan.

### Demographic history

Historical demographic changes of phylogenetic groups were examined based on Tajima’s *D*^71,72^, Fu’s *F*s^73^, mismatch distribution^74^ and an extended Bayesian skyline plot (EBSP)^75^. Tajima’s *D* and Fu’s *F*s were quantified using Arlequin 3.5^62^ by coalescent simulations under the infinite-sites model. The mismatch distribution for each phylogenetic group was analyzed to examine the possibility of sudden population expansion^76^ in Arlequin. Finally, EBSP were generated independently for three mitochondrial loci using BEAST 1.8.2^77^ to provide evidence of the historical expansion of geographic groups. BEAST was run for 60 million MCMC generations, with sampling every 5000th tree, based on TIM3 + I + G and a strict clock model (1% pairwise divergence per million years)^78^. TRACER 1.6^59^ was used to confirm whether ESS reached above 200 and the MCMC parameters of runs converged on the same stationary point with unimodal distribution.

## Electronic supplementary material

Supplementary material
