# Supplementary material for: The genetic structure of Squalidus multimaculatus revealing the historical pattern of serial colonization on the tip of East Asian continent
Source: Sci Rep. 2018 Jul 13;8:10629. doi: 10.1038/s41598-018-28340-x (PMC6045656; doi:10.1038/s41598-018-28340-x)
Supplement: Supplementary file 1 — Supplementary material [file 41598_2018_28340_MOESM1_ESM.docx]

The genetic structure of *Squalidus multimaculatus* revealing the historical pattern of serial colonization on the tip of East Asian continent.

Hyung-Bae Jeon, Dong-Young Kim, Yoon Jeong Lee, Han-Gyu Bae and Ho Young Suk*

Department of Life Sciences, Yeungnam University, 280 Daehak-ro, Gyeongsan, Gyeongsangbuk-do 38541, South Korea

* Corresponding author, E-mail: hsuk@ynu.ac.kr

Resubmitted to *Scientific Reports*

May 08, 2018

Supplementary Figure Legends

**Figure S1.** Sequence analysis of RAG1 gene from *Squalidus* species. Red arrows show the occurrence of multiple peaks in hybrid individuals between *S. multimaculatus* (SM) and *S. gracilis majimae* (SG).

**Figure S2.** Neighbor-joining (NJ) phylogenetic relationships of cyt *b* haplotypes from *Squalidus multimaculatus* and *S. gracilis majimae*. Node supporting values estimated from NJ, maximum likelihood (ML) and Bayesian inference (BI; converted to the 100^th^ percentile) algorithms were indicated on the branches. Three algorithms generated highly similar trees in branching pattern.

**Figure S3.** Bayesian inference (BI) phylogenetic relationships of COI haplotypes from *Squalidus multimaculatus* and *S. gracilis majimae*. Node supporting values estimated from BI (converted to the 100^th^ percentile) and maximum likelihood (ML) algorithms were indicated on the branches; only BI supporting values were indicated in lower nodes. Neighbor-joining (NJ) algorithm was not included because of the difference in branching pattern.

**Figure S4.** Bayesian inference (BI) phylogenetic relationships of 12*S* haplotypes from *Squalidus multimaculatus* and *S. gracilis majimae*. Node supporting values estimated from BI (converted to the 100^th^ percentile) and maximum likelihood (ML) algorithms were indicated on the branches; only BI supporting values were indicated in lower nodes. Neighbor-joining (NJ) algorithm was not included because of the difference in branching pattern.

**Figure S5.** Unrooted haplotype networks generated based on the 12*S* (A), COI (B) and cyt *b* (C) from *Squalidus multimaculatus* and *S. gracilis majimae*. See Figure 1 for the details about how to generate map.

**Figure S1.**


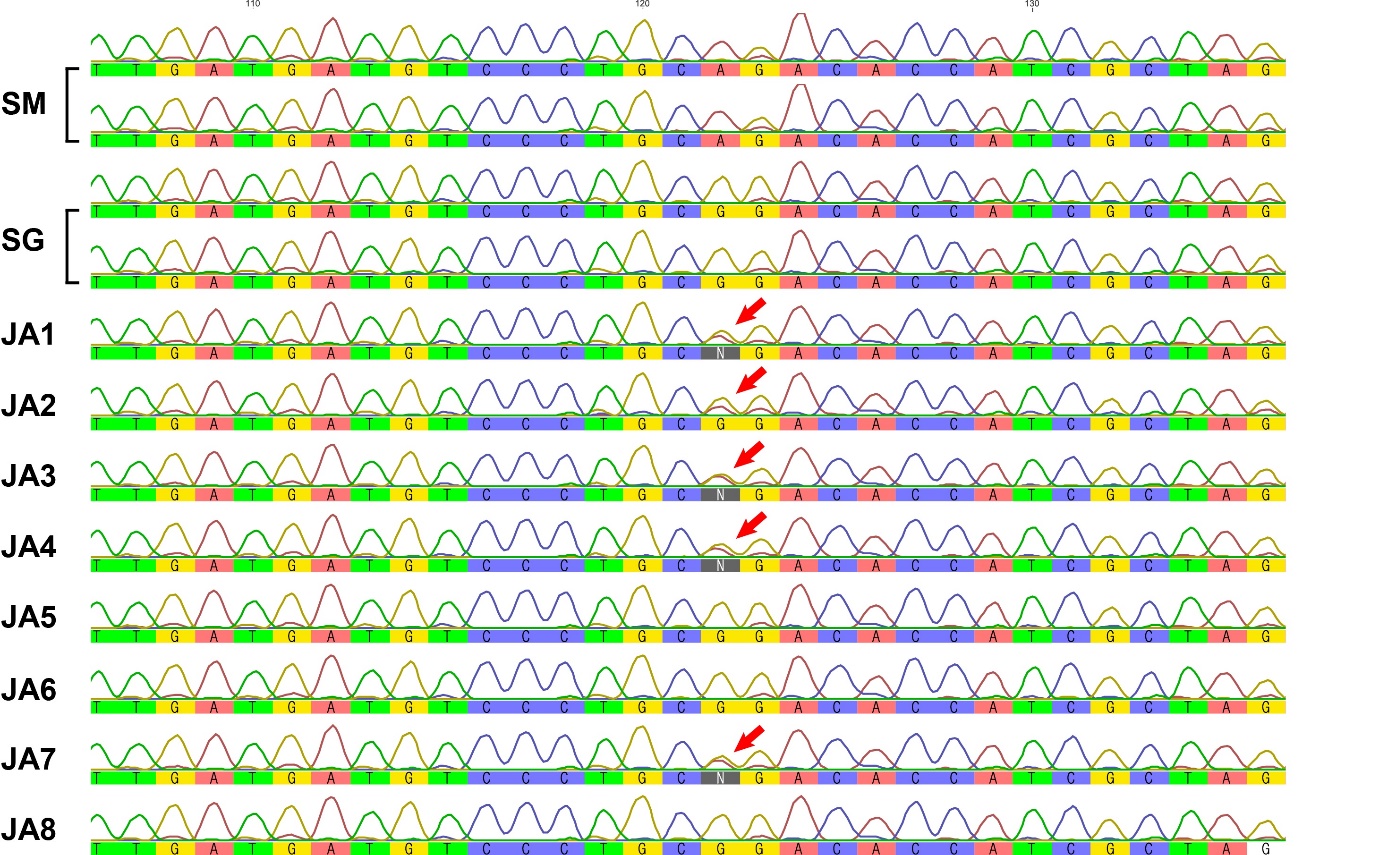


**Figure S2.**

**
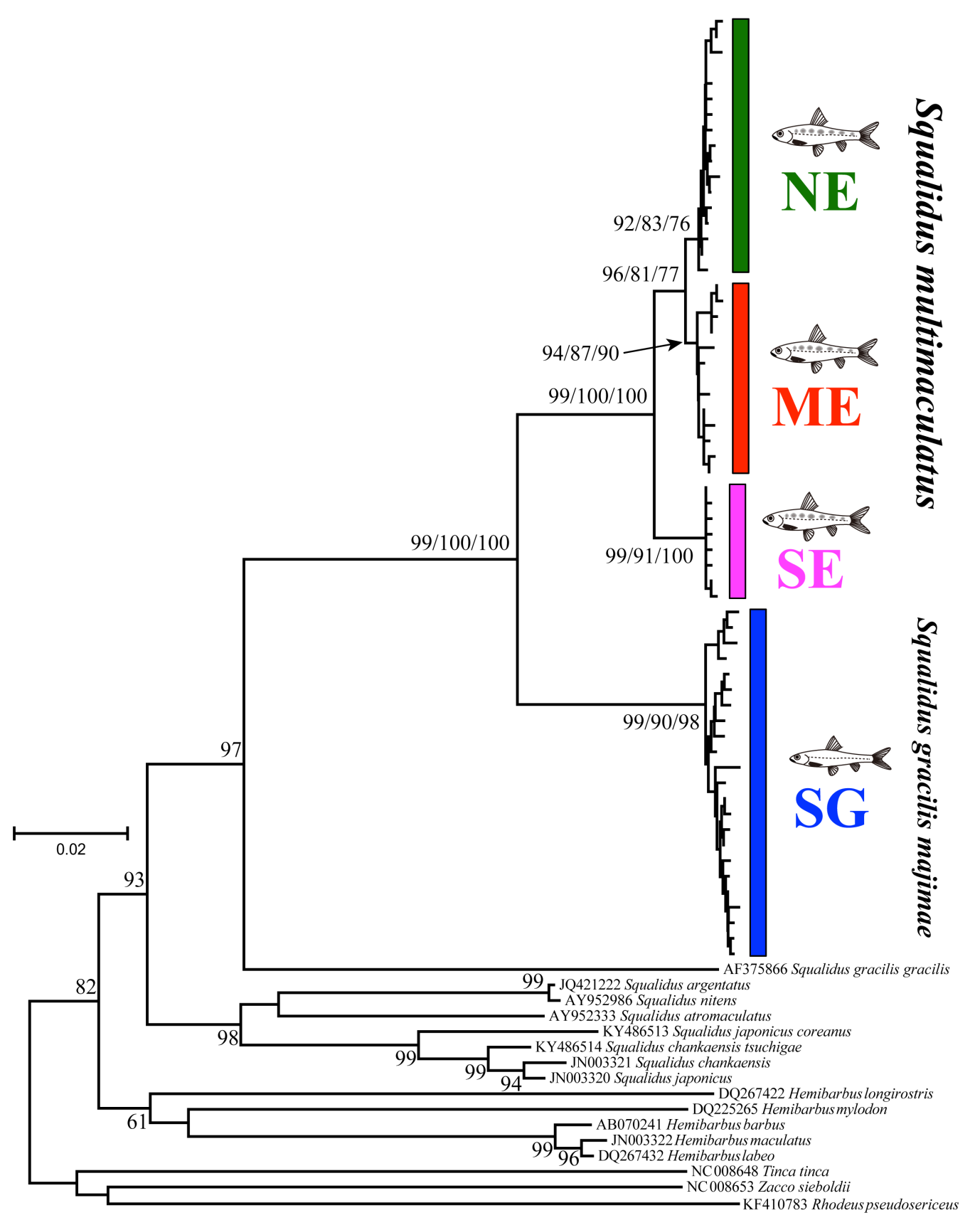
**

**Figure S3.**

**
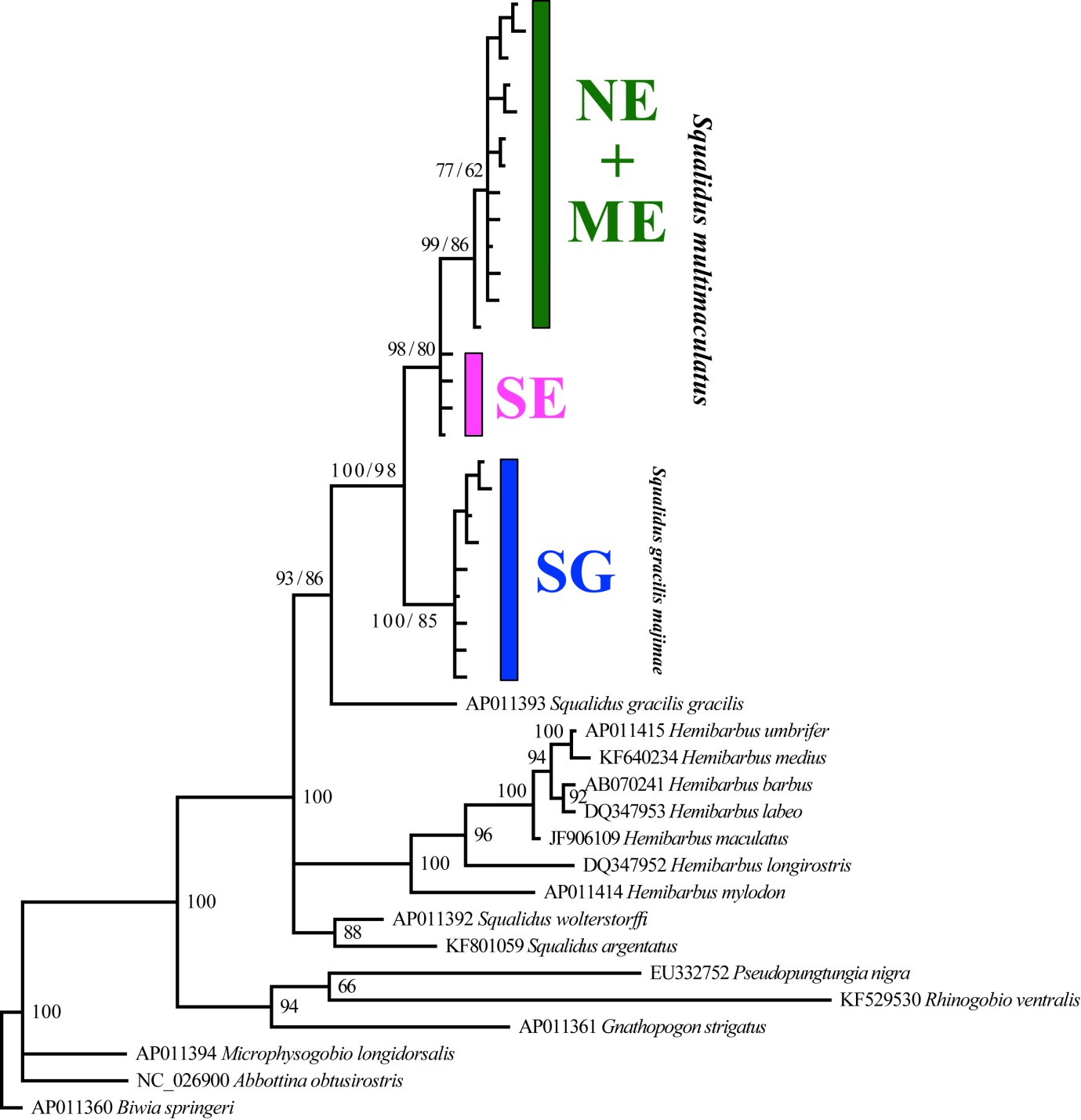
**

**Figure S4.**

**
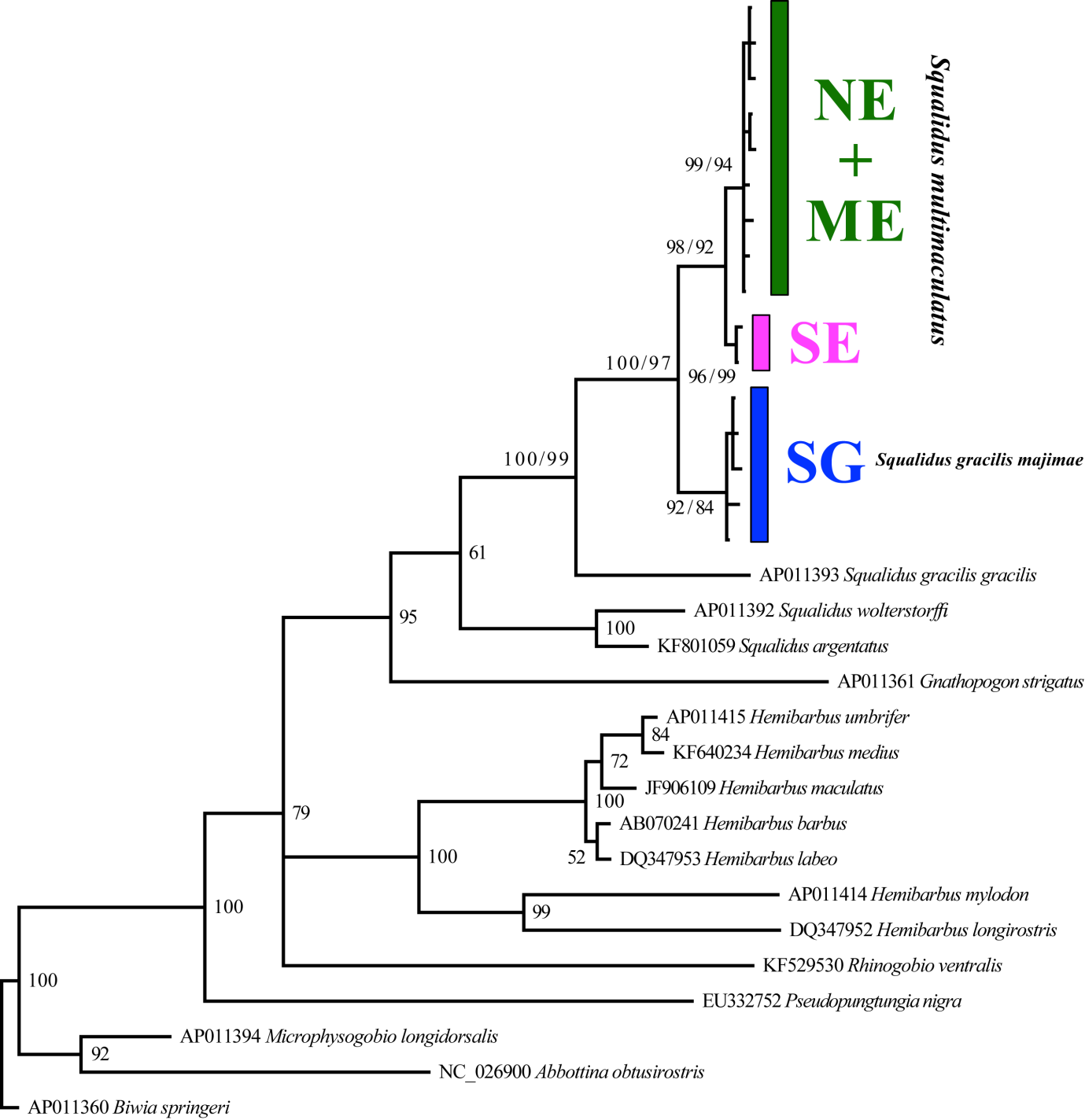
**

**Figure S5.**


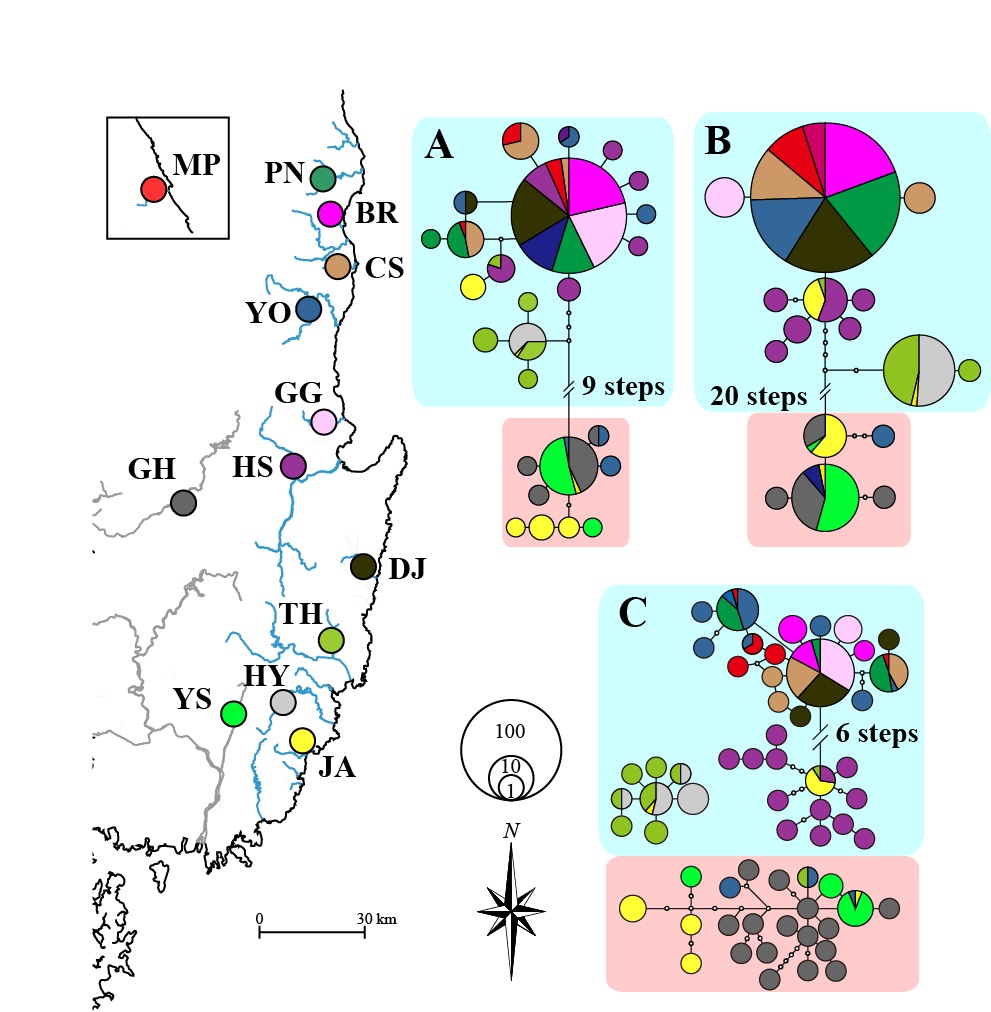


**Table S1.** The 13 sampling sites of *Squalidus multimaculatus* (SM) and *S. gracilis majimae* (SG) from the Korean Peninsula.

| Drainage | Population  ID | *N* | Latitude | Longitude | Species |
| --- | --- | --- | --- | --- | --- |
| Myeongpa River | MP | 9 | 38° 52' 79" | 128° 39' 82" | SM |
| Pyeonghae-Namdae River | PN | 20 | 36° 72' 11" | 129° 44' 23" | SM |
| Baegrok River | BR | 20 | 36° 59' 69" | 129° 40' 05" | SM |
| Chuksan River | CS | 20 | 36° 49' 82" | 129° 43' 39" | SM |
| Youngdeok-Osip River | YO | 20 | 36° 41' 76" | 129° 36' 39" | SM+SG |
| Gokgang River | GG | 20 | 36° 12' 28" | 129° 34' 54" | SM |
| Hyeongsan River | HS | 20 | 35° 81' 98" | 129° 28' 41" | SM |
| Daejong River | DJ | 20 | 35° 75' 03" | 129° 46' 57" | SM |
| Taehwa River | TH | 20 | 35° 56' 18" | 129° 26' 87" | SM |
| Hoeya River | HY | 20 | 35° 43' 72" | 129° 24' 27" | SM |
| Jangan River | JA | 20 | 35° 33' 54" | 129° 26' 86" | SM+SG |
| Yangsan Tributary (Nakdong River) | YS | 20 | 35° 28' 03" | 129° 04' 49" | SG |
| Geumho Tributary (Nakdong River) | GH | 20 | 35° 43' 73" | 128° 97' 91'' | SG |

**Table S2.** COI haplotypes obtained in this study and the frequency distribution throughout the collection sites.

| Haplotype | MP | PN | BR | CS | YO | GG | HS | DJ | TH | HY | JA | YS | GH |
| --- | --- | --- | --- | --- | --- | --- | --- | --- | --- | --- | --- | --- | --- |
| NE01 | 9 | 20 | 20 | 12 | 16 | 5 |  | 20 |  |  |  |  |  |
| NE02 |  |  |  | 8 |  |  |  |  |  |  |  |  |  |
| NE03 |  |  |  |  |  | 15 |  |  |  |  |  |  |  |
| ME01 |  |  |  |  |  |  | 1 |  |  |  |  |  |  |
| ME02 |  |  |  |  |  |  | 5 |  |  |  |  |  |  |
| ME03 |  |  |  |  |  |  | 2 |  |  |  |  |  |  |
| ME04 |  |  |  |  |  |  | 1 |  |  |  |  |  |  |
| ME05 |  |  |  |  |  |  | 1 |  |  |  |  |  |  |
| ME06 |  |  |  |  |  |  | 10 |  | 1 |  | 7 |  |  |
| SE01 |  |  |  |  |  |  |  |  | 1 |  |  |  |  |
| SE02 |  |  |  |  |  |  |  |  | 18 | 20 | 1 |  |  |
| GM01 |  |  |  |  | 1 |  |  |  |  |  |  |  |  |
| GM02 |  |  |  |  |  |  |  |  |  |  | 11 | 1 | 6 |
| GM03 |  |  |  |  | 3 |  |  |  |  |  | 1 | 19 | 12 |
| GM04 |  |  |  |  |  |  |  |  |  |  |  |  | 1 |
| GM05 |  |  |  |  |  |  |  |  |  |  |  |  | 1 |
|  | 1 | 1 | 1 | 2 | 3 | 2 | 6 | 1 | 3 | 1 | 4 | 2 | 4 |

**Table S3.** Cyt *b* haplotypes obtained in this study and the frequency distribution throughout the collection sites.

| Haplotype | MP | PN | BR | CS | YO | GG | HS | DJ | TH | HY | JA | YS | GH |
| --- | --- | --- | --- | --- | --- | --- | --- | --- | --- | --- | --- | --- | --- |
| NE01 |  | 2 | 6 | 10 |  | 16 |  | 13 |  |  |  |  |  |
| NE02 | 1 | 9 |  |  | 10 |  |  | 2 |  |  |  |  |  |
| NE03 | 1 | 9 |  | 8 | 1 |  |  |  |  |  |  |  |  |
| NE04 | 1 |  |  |  |  |  |  |  |  |  |  |  |  |
| NE05 | 2 |  |  |  | 1 |  |  |  |  |  |  |  |  |
| NE06 | 4 |  |  |  |  |  |  |  |  |  |  |  |  |
| NE07 |  |  | 9 |  |  |  |  |  |  |  |  |  |  |
| NE08 |  |  | 5 |  |  |  |  |  |  |  |  |  |  |
| NE09 |  |  |  | 1 |  |  |  |  |  |  |  |  |  |
| NE10 |  |  |  | 1 |  |  |  |  |  |  |  |  |  |
| NE11 |  |  |  |  |  |  |  | 2 |  |  |  |  |  |
| NE12 |  |  |  |  |  |  |  | 3 |  |  |  |  |  |
| NE13 |  |  |  |  |  | 4 |  |  |  |  |  |  |  |
| NE14 |  |  |  |  | 1 |  |  |  |  |  |  |  |  |
| NE15 |  |  |  |  | 1 |  |  |  |  |  |  |  |  |
| NE16 |  |  |  |  | 1 |  |  |  |  |  |  |  |  |
| NE17 |  |  |  |  | 1 |  |  |  |  |  |  |  |  |
| ME01 |  |  |  |  |  |  | 2 |  |  |  |  |  |  |
| ME02 |  |  |  |  |  |  | 1 |  |  |  |  |  |  |
| ME03 |  |  |  |  |  |  | 3 |  | 1 |  | 7 |  |  |
| ME04 |  |  |  |  |  |  | 2 |  |  |  |  |  |  |
| ME05 |  |  |  |  |  |  | 2 |  |  |  |  |  |  |
| ME06 |  |  |  |  |  |  | 1 |  |  |  |  |  |  |
| ME07 |  |  |  |  |  |  | 1 |  |  |  |  |  |  |
| ME08 |  |  |  |  |  |  | 1 |  |  |  |  |  |  |
| ME09 |  |  |  |  |  |  | 1 |  |  |  |  |  |  |
| ME10 |  |  |  |  |  |  | 2 |  |  |  |  |  |  |
| ME11 |  |  |  |  |  |  | 1 |  |  |  |  |  |  |
| ME12 |  |  |  |  |  |  | 1 |  |  |  |  |  |  |
| ME13 |  |  |  |  |  |  | 2 |  |  |  |  |  |  |
| SE01 |  |  |  |  |  |  |  |  |  | 12 |  |  |  |
| SE02 |  |  |  |  |  |  |  |  | 1 | 1 |  |  |  |
| SE03 |  |  |  |  |  |  |  |  | 1 |  |  |  |  |
| SE04 |  |  |  |  | 1 |  |  |  | 5 |  |  |  |  |
| SE05 |  |  |  |  |  |  |  |  | 4 |  |  |  |  |
| SE06 |  |  |  |  |  |  |  |  | 1 |  |  |  |  |
| SE07 |  |  |  |  |  |  |  |  | 1 |  |  |  |  |
| SE08 |  |  |  |  |  |  |  |  | 5 | 7 | 1 |  |  |
| GM01 |  |  |  |  | 1 |  |  |  | 1 |  |  |  |  |
| GM02 |  |  |  |  |  |  |  |  |  |  | 8 |  |  |
| GM03 |  |  |  |  |  |  |  |  |  |  | 2 |  |  |
| GM04 |  |  |  |  |  |  |  |  |  |  | 1 |  |  |
| GM05 |  |  |  |  |  |  |  |  |  |  |  |  | 1 |
| GM06 |  |  |  |  |  |  |  |  |  |  |  |  | 1 |
| GM07 |  |  |  |  |  |  |  |  |  |  |  |  | 2 |
| GM08 |  |  |  |  |  |  |  |  |  |  |  |  | 1 |
| GM09 |  |  |  |  |  |  |  |  |  |  |  |  | 2 |
| GM10 |  |  |  |  |  |  |  |  |  |  |  |  | 1 |
| GM11 |  |  |  |  |  |  |  |  |  |  |  |  | 1 |
| GM12 |  |  |  |  |  |  |  |  |  |  |  |  | 3 |
| GM13 |  |  |  |  |  |  |  |  |  |  |  |  | 1 |
| GM14 |  |  |  |  |  |  |  |  |  |  |  |  | 1 |
| GM15 |  |  |  |  |  |  |  |  |  |  |  |  | 1 |
| GM16 |  |  |  |  |  |  |  |  |  |  |  |  | 1 |
| GM17 |  |  |  |  |  |  |  |  |  |  |  |  | 1 |
| GM18 |  |  |  |  |  |  |  |  |  |  |  |  | 2 |
| GM19 |  |  |  |  |  |  |  |  |  |  |  |  | 1 |
| GM20 |  |  |  |  | 1 |  |  |  |  |  | 1 | 14 |  |
| GM21 |  |  |  |  |  |  |  |  |  |  |  | 5 |  |
| GM22 |  |  |  |  |  |  |  |  |  |  |  | 1 |  |
| GM23 |  |  |  |  | 1 |  |  |  |  |  |  |  |  |
|  | 5 | 3 | 3 | 4 | 11 | 2 | 13 | 4 | 9 | 3 | 6 | 3 | 15 |

**Table S4.** 12*S* haplotypes obtained in this study and the frequency distribution throughout the collection sites.

| Haplotype | MP | PN | BR | CS | YO | GG | HS | DJ | TH | HY | JA | YS | GH |
| --- | --- | --- | --- | --- | --- | --- | --- | --- | --- | --- | --- | --- | --- |
| NE01 | 4 | 11 | 20 | 2 | 11 | 20 | 7 | 18 |  |  |  |  |  |
| NE02 | 4 |  |  | 10 |  |  |  |  |  |  |  |  |  |
| NE03 | 1 | 8 |  | 8 |  |  |  |  |  |  |  |  |  |
| NE04 |  | 1 |  |  |  |  |  |  |  |  |  |  |  |
| NE05 |  |  |  |  | 1 |  |  |  |  |  |  |  |  |
| NE06 |  |  |  |  | 2 |  |  | 2 |  |  |  |  |  |
| ME01 |  |  |  |  | 2 |  | 1 |  |  |  |  |  |  |
| ME02 |  |  |  |  |  |  | 5 |  |  |  |  |  |  |
| ME03 |  |  |  |  |  |  | 4 |  | 1 |  |  |  |  |
| ME04 |  |  |  |  |  |  | 1 |  |  |  |  |  |  |
| ME05 |  |  |  |  |  |  | 1 |  |  |  |  |  |  |
| ME06 |  |  |  |  |  |  | 1 |  |  |  |  |  |  |
| NE12 |  |  |  |  |  |  |  |  |  |  | 7 |  |  |
| SE01 |  |  |  |  |  |  |  |  | 1 |  |  |  |  |
| SE02 |  |  |  |  |  |  |  |  | 6 |  |  |  |  |
| SE03 |  |  |  |  |  |  |  |  | 1 |  |  |  |  |
| SE04 |  |  |  |  |  |  |  |  | 11 | 20 | 1 |  |  |
| GM01 |  |  |  |  | 2 |  |  |  |  |  |  |  |  |
| GM02 |  |  |  |  |  |  |  |  |  |  | 7 |  |  |
| GM03 |  |  |  |  |  |  |  |  |  |  | 1 |  |  |
| GM04 |  |  |  |  |  |  |  |  |  |  | 3 |  |  |
| GM05 |  |  |  |  | 1 |  |  |  |  |  | 1 | 19 | 16 |
| GM06 |  |  |  |  |  |  |  |  |  |  |  |  | 1 |
| GM07 |  |  |  |  |  |  |  |  |  |  |  |  | 2 |
| GM08 |  |  |  |  | 1 |  |  |  |  |  |  |  | 1 |
| GM09 |  |  |  |  |  |  |  |  |  |  |  | 1 |  |
|  | 3 | 3 | 1 | 3 | 7 | 1 | 7 | 2 | 5 | 1 | 6 | 2 | 4 |

**Table S5.** Haplotypes obtained from the combined loci (COI + cyt *b* + 12S) data in this study and the frequency distribution throughout the collection sites.

| Haplotype | MP | PN | BR | CS | YO | GG | HS | DJ | TH | HY | JA | YS | GH |
| --- | --- | --- | --- | --- | --- | --- | --- | --- | --- | --- | --- | --- | --- |
| NE1 | 1 | 9 |  |  | 7 |  |  | 2 |  |  |  |  |  |
| NE2 |  | 2 | 6 |  |  | 1 |  | 13 |  |  |  |  |  |
| NE3 |  |  | 9 |  |  |  |  |  |  |  |  |  |  |
| NE4 |  |  | 4 |  |  |  |  |  |  |  |  |  |  |
| NE5 | 1 |  |  |  |  |  |  |  |  |  |  |  |  |
| NE6 | 1 | 8 |  |  |  |  |  |  |  |  |  |  |  |
| NE7 | 2 |  |  |  | 1 |  |  |  |  |  |  |  |  |
| NE8 | 4 |  |  |  |  |  |  |  |  |  |  |  |  |
| NE9 |  | 1 |  |  |  |  |  |  |  |  |  |  |  |
| NE10 |  |  |  | 10 |  |  |  |  |  |  |  |  |  |
| NE11 |  |  |  | 8 |  |  |  |  |  |  |  |  |  |
| NE12 |  |  |  | 1 |  |  |  |  |  |  |  |  |  |
| NE13 |  |  |  | 1 |  |  |  |  |  |  |  |  |  |
| NE14 |  |  |  |  | 1 |  |  |  |  |  |  |  |  |
| NE15 |  |  |  |  | 1 |  |  |  |  |  |  |  |  |
| NE16 |  |  |  |  | 1 |  |  |  |  |  |  |  |  |
| NE17 |  |  |  |  | 1 |  |  |  |  |  |  |  |  |
| NE18 |  |  |  |  | 1 |  |  |  |  |  |  |  |  |
| NE19 |  |  |  |  | 1 |  |  |  |  |  |  |  |  |
| NE20 |  |  |  |  | 1 |  |  |  |  |  |  |  |  |
| NE21 |  |  |  |  | 1 |  |  |  |  |  |  |  |  |
| NE22 |  |  |  |  |  | 15 |  |  |  |  |  |  |  |
| NE23 |  |  |  |  |  | 4 |  |  |  |  |  |  |  |
| NE24 |  |  |  |  |  |  |  | 2 |  |  |  |  |  |
| NE25 |  |  |  |  |  |  |  | 3 |  |  |  |  |  |
| HS1 |  |  |  |  |  |  | 2 |  |  |  |  |  |  |
| HS2 |  |  |  |  |  |  | 1 |  |  |  |  |  |  |
| HS3 |  |  |  |  |  |  | 3 |  | 1 |  |  |  |  |
| HS4 |  |  |  |  |  |  | 2 |  |  |  |  |  |  |
| HS5 |  |  |  |  |  |  | 2 |  |  |  |  |  |  |
| HS6 |  |  |  |  |  |  | 1 |  |  |  |  |  |  |
| HS7 |  |  |  |  |  |  | 1 |  |  |  |  |  |  |
| HS8 |  |  |  |  |  |  | 1 |  |  |  |  |  |  |
| HS9 |  |  |  |  |  |  | 1 |  |  |  |  |  |  |
| HS10 |  |  |  |  |  |  | 1 |  |  |  |  |  |  |
| HS11 |  |  |  |  |  |  | 1 |  |  |  |  |  |  |
| HS12 |  |  |  |  |  |  | 1 |  |  |  |  |  |  |
| HS13 |  |  |  |  |  |  | 1 |  |  |  |  |  |  |
| HS14 |  |  |  |  |  |  | 1 |  |  |  |  |  |  |
| HS15 |  |  |  |  |  |  | 1 |  |  |  |  |  |  |
| HS16 |  |  |  |  |  |  |  |  |  |  | 7 |  |  |
| SE1 |  |  |  |  |  |  |  |  | 1 |  |  |  |  |
| SE2 |  |  |  |  |  |  |  |  | 1 |  |  |  |  |
| SE3 |  |  |  |  |  |  |  |  | 4 |  |  |  |  |
| SE4 |  |  |  |  |  |  |  |  | 1 |  |  |  |  |
| SE5 |  |  |  |  |  |  |  |  | 4 |  |  |  |  |
| SE6 |  |  |  |  |  |  |  |  | 1 |  |  |  |  |
| SE7 |  |  |  |  |  |  |  |  | 1 |  |  |  |  |
| SE8 |  |  |  |  |  |  |  |  | 1 |  |  |  |  |
| SE9 |  |  |  |  |  |  |  |  | 1 |  |  |  |  |
| SE10 |  |  |  |  |  |  |  |  |  | 12 |  |  |  |
| SE11 |  |  |  |  |  |  |  |  | 2 | 7 | 1 |  |  |
| SE12 |  |  |  |  |  |  |  |  | 1 | 1 |  |  |  |
| SG1 |  |  |  |  |  |  |  |  |  |  | 7 |  |  |
| SG2 |  |  |  |  |  |  |  |  |  |  | 1 |  |  |
| SG3 |  |  |  |  |  |  |  |  |  |  | 2 |  |  |
| SG4 |  |  |  |  | 1 |  |  |  |  |  | 1 | 14 |  |
| SG5 |  |  |  |  | 1 |  |  |  |  |  |  |  |  |
| SG6 |  |  |  |  | 1 |  |  |  |  |  |  |  |  |
| SG7 |  |  |  |  |  |  |  |  |  |  | 1 |  |  |
| SG8 |  |  |  |  |  |  |  |  |  |  |  | 5 |  |
| SG9 |  |  |  |  |  |  |  |  |  |  |  | 1 |  |
| SG10 |  |  |  |  |  |  |  |  |  |  |  |  | 1 |
| SG11 |  |  |  |  |  |  |  |  |  |  |  |  | 1 |
| SG12 |  |  |  |  |  |  |  |  |  |  |  |  | 2 |
| SG13 |  |  |  |  |  |  |  |  |  |  |  |  | 1 |
| SG14 |  |  |  |  |  |  |  |  |  |  |  |  | 2 |
| SG15 |  |  |  |  |  |  |  |  |  |  |  |  | 1 |
| SG16 |  |  |  |  |  |  |  |  |  |  |  |  | 1 |
| SG17 |  |  |  |  |  |  |  |  |  |  |  |  | 3 |
| SG18 |  |  |  |  |  |  |  |  |  |  |  |  | 1 |
| SG19 |  |  |  |  |  |  |  |  |  |  |  |  | 1 |
| SG20 |  |  |  |  |  |  |  |  |  |  |  |  | 1 |
| SG21 |  |  |  |  |  |  |  |  |  |  |  |  | 1 |
| SG22 |  |  |  |  |  |  |  |  |  |  |  |  | 1 |
| SG23 |  |  |  |  |  |  |  |  |  |  |  |  | 2 |
| SG24 |  |  |  |  |  |  |  |  |  |  |  |  | 1 |
| SG25 |  |  |  |  |  |  |  |  | 1 |  |  |  |  |
|  | 4 | 3 | 3 | 4 | 12 | 3 | 15 | 3 | 12 | 3 | 7 | 3 | 15 |

**Table S6.** Summary of the AMOVA results based on three mitochondrial loci that partition genetic variation among *S. multimaculatus* and *S. g. majimae* populations from the Korean Peninsula to three different hierarchical levels.

| Source of variation | d.f. | Percentage of variation |
| --- | --- | --- |
| Among groups | 3 | 77.72 |
| Among populations | 9 | 6.39 |
| Within populations | 236 | 15.89 |
| Total | 248 | 100 |

**Table S7.** The genetic diversity indices of the *Squalidus multimaculatus* and *S. gracilis majimae* estimated based on mitochondrial COI sequences. Data comprise number of individuals analyzed (*N*), nucleotide diversity (*π*), number of haplotypes (*h*), haplotype diversity *(h_d_*), number of segregating sites (*S*), Tajima’s *D*, Fu’s *F*s and sum of squares deviation (SSD). Statistically significant values were highlighted with bold.

| Pop (haplogroup) | *N* | *π* | *h* | *h_d_* | *S* | Tajima's *D* | Fu's *F*s | SSD |
| --- | --- | --- | --- | --- | --- | --- | --- | --- |
| MP (NE) | 9 | 0.00000 | 1 | 0 | 0 | 0.000 | 0.000 | 0.000 |
| PN (NE) | 20 | 0.00000 | 1 | 0 | 0 | 0.000 | 0.000 | 0.000 |
| BR (NE) | 20 | 0.00000 | 1 | 0 | 0 | 0.000 | 0.000 | 0.000 |
| CS (NE) | 20 | 0.00075 | 2 | 0.505 | 1 | 1.430 | 1.409 | 0.023 |
| YO (NE) | 20 | 0.01342 | 3 | 0.353 | 29 | 0.390 | 12.243 | **0.119** |
| GG (NE) | 20 | 0.00059 | 2 | 0.395 | 1 | 0.722 | 0.976 | 0.006 |
| HS (ME) | 20 | 0.00167 | 6 | 0.705 | 6 | -1.074 | -1.915 | 0.001 |
| DJ (NE) | 20 | 0.00000 | 1 | 0 | 0 | 0.000 | 0.000 | 0.000 |
| TH (SE) | 20 | 0.00104 | 3 | 0.195 | 7 | **-2.121** | 0.443 | 0.012 |
| HY (SE) | 20 | 0.00000 | 1 | 0 | 0 | 0.000 | 0.000 | 0.000 |
| JA (ME) | 20 | 0.01839 | 4 | 0.600 | 27 | 2.409 | 12.280 | 0.234 |
| YA | 20 | 0.00015 | 2 | 0.100 | 1 | -1.164 | -0.879 | 0.001 |
| GH | 20 | 0.00111 | 4 | 0.574 | 4 | -0.989 | -0.683 | 0.009 |

**Table S8.** The genetic diversity indices of the *Squalidus multimaculatus* and *S. gracilis majimae* estimated based on mitochondrial cyt *b* sequences. Data comprise number of individuals analyzed (*N*), nucleotide diversity (*π*), number of haplotypes (*h*), haplotype diversity *(h_d_*), number of segregating sites (*S*), Tajima’s *D*, Fu’s *F*s and sum of squares deviation (SSD). Statistically significant values were highlighted with bold.

| Pop (haplogroup) | *N* | *π* | *h* | *h_d_* | *S* | Tajima's *D* | Fu's *F*s | SSD |
| --- | --- | --- | --- | --- | --- | --- | --- | --- |
| MP (NE) | 9 | 0.00158 | 5 | 0.806 | 6 | -0.849 | -1.113 | 0.002 |
| PN (NE) | 20 | 0.00139 | 3 | 0.616 | 3 | 2.266 | 2.795 | 0.118 |
| BR (NE) | 20 | 0.00081 | 3 | 0.679 | 2 | 1.464 | 1.020 | 0.007 |
| CS (NE) | 20 | 0.00115 | 4 | 0.616 | 4 | 0.443 | 0.617 | 0.130 |
| YO (NE) | 20 | 0.01984 | 11 | 0.763 | 94 | -0.673 | 3.907 | 0.035 |
| GG (NE) | 20 | 0.00030 | 2 | 0.337 | 1 | 0.352 | 0.721 | 0.003 |
| HS (ME) | 20 | 0.00354 | 13 | 0.958 | 18 | -0.804 | **-4.984** | 0.004 |
| DJ (NE) | 20 | 0.00091 | 4 | 0.563 | 5 | -0.832 | 0.051 | **0.167** |
| TH (SE) | 20 | 0.00972 | 9 | 0.863 | 93 | **-2.424** | 2.795 | 0.028 |
| HY (SE) | 20 | 0.00054 | 3 | 0.542 | 2 | 0.173 | 0.153 | 0.021 |
| JA (ME) | 20 | 0.03611 | 6 | 0.737 | 92 | 2.205 | 19.169 | 0.232 |
| YA | 20 | 0.00095 | 3 | 0.468 | 7 | -1.773 | 1.381 | 0.022 |
| GH | 20 | 0.00347 | 15 | 0.968 | 24 | **-1.625** | **-8.337** | 0.004 |

**Table S9.** The genetic diversity indices of the *Squalidus multimaculatus* and *S. gracilis majimae* estimated based on mitochondrial 12S sequences. Data comprise number of individuals analyzed (*N*), nucleotide diversity (*π*), number of haplotypes (*h*), haplotype diversity *(h_d_*), number of segregating sites (*S*), Tajima’s *D*, Fu’s *F*s and sum of squares deviation (SSD). Statistically significant values were highlighted with bold.

| Pop (haplogroup) | *N* | *π* | *h* | *h_d_* | *S* | Tajima's *D* | Fu's *F*s | SSD |
| --- | --- | --- | --- | --- | --- | --- | --- | --- |
| MP (NE) | 9 | 0.00105 | 3 | 0.667 | 3 | -0.359 | 0.351 | 0.014 |
| PN (NE) | 20 | 0.00120 | 3 | 0.563 | 3 | 0.936 | 1.546 | 0.136 |
| BR (NE) | 20 | 0.00000 | 1 | 0 | 0 | 0.000 | 0.000 | 0.000 |
| CS (NE) | 20 | 0.00162 | 3 | 0.611 | 3 | 2.183 | 2.358 | 0.116 |
| YO (NE) | 20 | 0.00538 | 7 | 0.695 | 17 | 0.244 | 1.726 | **0.084** |
| GG (NE) | 20 | 0.00000 | 1 | 0 | 0 | 0.000 | 0.000 | 0.000 |
| HS (ME) | 20 | 0.00155 | 7 | 0.805 | 7 | -0.840 | -2.183 | 0.002 |
| DJ (NE) | 20 | 0.00020 | 2 | 0.189 | 1 | -0.591 | -0.096 | 0.015 |
| TH (SE) | 20 | 0.00141 | 5 | 0.563 | 10 | **-1.831** | -0.364 | 0.027 |
| HY (SE) | 20 | 0.00000 | 1 | 0 | 0 | 0.000 | 0.000 | 0.000 |
| JA (ME) | 20 | 0.01017 | 6 | 0.763 | 21 | 2.403 | 6.084 | **0.108** |
| YA | 20 | 0.00032 | 2 | 0.100 | 3 | **-1.723** | 0.543 | 0.014 |
| GH | 20 | 0.00041 | 4 | 0.363 | 3 | -1.440 | **-2.135** | 0.003 |

**Table S10.** The comparison of genetic diversity indices among the haplogroups of *Squalidus multimaculatus* and *S. gracilis majimae* estimated based on mitochondrial COI sequences. Data comprise number of individuals analyzed (*N*), nucleotide diversity (*π*), number of haplotypes (*h*), haplotype diversity *(h_d_*), number of segregating sites (*S*), Tajima’s *D*, Fu’s *F*s and sum of squares deviation (SSD). Statistically significant values were highlighted with bold.

| Haplogroup | *N* | *π* | *h* | *h_d_* | *S* | Tajima's *D* | Fu's *F*s | SSD |
| --- | --- | --- | --- | --- | --- | --- | --- | --- |
| NE | 125 | 0.00050 | 3 | 0.318 | 2 | -0.148 | -0.001 | 0.002 |
| ME | 29 | 0.00183 | 7 | 0.596 | 12 | **-1.967** | -2.082 | 0.003 |
| SE | 40 | 0.00179 | 3 | 0.099 | 24 | **-2.655** | 2.440 | 0.003 |
| SG | 55 | 0.00101 | 5 | 0.519 | 6 | -1.207 | **-**1.087 | 0.015 |

**Table S11.** The comparison of genetic diversity indices among the haplogroups of *Squalidus multimaculatus* and *S. gracilis majimae* estimated based on mitochondrial cyt *b* sequences. Data comprise number of individuals analyzed (*N*), nucleotide diversity (*π*), number of haplotypes (*h*), haplotype diversity *(h_d_*), number of segregating sites (*S*), Tajima’s *D*, Fu’s *F*s and sum of squares deviation (SSD). Statistically significant values were highlighted with bold.

| Haplogroup | *N* | *π* | *h* | *h_d_* | *S* | Tajima's *D* | Fu's *F*s | SSD |
| --- | --- | --- | --- | --- | --- | --- | --- | --- |
| NE | 125 | 0.00139 | 17 | 0.800 | 17 | -1.382 | **18.206** | 0.001 |
| ME | 29 | 0.00268 | 13 | 0.841 | 18 | -1.211 | **-4.541** | 0.005 |
| SE | 40 | 0.00453 | 9 | 0.803 | 86 | **-2.723** | 2.090 | **0.023** |
| SG | 55 | 0.00395 | 23 | 0.889 | 36 | -1.498 | **-8.633** | 0.014 |

**Table S12.** The comparison of genetic diversity indices among the haplogroups of *Squalidus multimaculatus* and *S. gracilis majimae* estimated based on mitochondrial 12S sequences. Data comprise number of individuals analyzed (*N*), nucleotide diversity (*π*), number of haplotypes (*h*), haplotype diversity *(h_d_*), number of segregating sites (*S*), Tajima’s *D*, Fu’s *F*s and sum of squares deviation (SSD). Statistically significant values were highlighted with bold.

| Haplogroup | *N* | *π* | *h* | *h_d_* | *S* | Tajima's *D* | Fu's *F*s | SSD |
| --- | --- | --- | --- | --- | --- | --- | --- | --- |
| NE | 125 | 0.00085 | 7 | 0.501 | 5 | -0.259 | -1.726 | 0.005 |
| ME | 29 | 0.00240 | 9 | 0.847 | 12 | -0.838 | -1.759 | 0.013 |
| SE | 40 | 0.00096 | 5 | 0.385 | 14 | **-2.281** | -0.578 | 0.006 |
| SG | 55 | 0.00130 | 9 | 0.534 | 9 | -1.021 | -2.965 | **0.370** |
